# Relationships Among the Rootstock, Crop Load, and Sugar Hormone Signaling of Apple Tree, and Their Effects on Biennial Bearing

**DOI:** 10.3389/fpls.2020.01213

**Published:** 2020-08-07

**Authors:** Darius Kviklys, Giedrė Samuolienė

**Affiliations:** ^1^ Lithuanian Research Centre for Agriculture and Forestry, Institute of Horticulture, Babtai, Lithuania; ^2^ Department of Horticulture, Norwegian Institute of Bioeconomy Research—NIBIO Ullensvang, Lofthus, Norway

**Keywords:** dwarfing rootstocks, *Malus* × *domestica* Borkh., phytohormones, return bloom, sugars

## Abstract

Adjustable crop load primarily involves bud manipulation, and usually switches from vegetative to reproductive buds. While this switch is not fully understood, it is still controlled by the ratio of hormones, which promote or inhibit bud formation. To determine the reasons for biennial bearing, the effect of apple rootstock, scion cultivar, crop load, as well as metabolic changes of endogenous phytohormones [zeatin, jasmonic acid, indole-3 acetic acid (IAA), abscisic acid (ABA), and gibberellins 1, 3, and 7 (GAs)], and soluble sugars (glucose, fructose, and sorbitol) were evaluated, and their connections with return bloom and yield of apple tree buds were analyzed. Cultivars “Ligol” and “Auksis” were tested on five rootstocks contrasting in induced vigor: semi-dwarfing M.26; dwarfing M.9, B.396, and P 67; and super-dwarfing P 22. Crop load levels were adjusted before flowering, leaving 75, 113, and 150 fruits per tree. Principal component analysis (PCA) scatter plot of the metabolic response of phytohormones and sugars indicated that the effect of the semi-dwarfing M.26 rootstock was significantly different from that of the dwarfing M.9 and P 67, as well as the super-dwarfing P 22 rootstocks in both varieties. The most intensive crop load (150 fruits per tree) produced a significantly different response compared to less intensive crop loads (113 and 75) in both varieties. In contrast to soluble sugar accumulation, increased crop load resulted in an increased accumulation of phytohormones, except for ABA. Dwarfing rootstocks M.9, B.396, and P 67, as well as super-dwarf P 22 produced an altered accumulation of promoter phytohormones, while the more vigorous semi-dwarfing M.26 rootstock induced a higher content of glucose and inhibitory phytohormones, by increasing content of IAA, ABA, and GAs. The most significant decrease in return bloom resulted from the highest crop load in “Auksis” grafted on M.9 and P 22 rootstocks. Average difference in flower number between crop loads of 75 and 150 fruits per tree in “Ligol” was 68%, while this difference reached ~ 90% for P 22, and ~ 75% for M.9 and M.26 rootstocks. Return bloom was dependent on the previous year’s crop load, cultivar, and rootstock.

## Introduction

Apple rootstocks are categorized according to tree vigor, i.e., dwarfing, semi-dwarfing, semi-vigorous, and vigorous. As the production of dwarfing rootstocks has greatly increased the yield efficiency, fruit size, and quality of the commercial apple orchard, focus on rootstock-scion interactions have become increasingly important. Studies performed in Lithuania investigating the effect of rootstock on fruit quality, particularly with respect to bioactive compound accumulation, revealed that a significantly higher phenol content was found in “Ligol” fruits on P 61 and P 22 rootstocks, compared to “Auksis” fruits produced on P 67 rootstock (Kviklys et al., 2014; Kviklys et al., 2017). Rootstock-cultivar interaction was found to be significant in evaluating the effect of rootstock on apple tree growth habit, or root architecture (Tworkoski and Miller, 2007; Harrison et al., 2016; Lordan et al., 2017).

The effect of different rootstocks on apple tree productivity was established in our rootstock trials performed in three countries. M.9 rootstock was the most productive under Lithuanian climate conditions; Pure 1, P 60, and B.9 were most productive in Latvia and Bulboga, while B.146 and M.26 were the most productive in Estonia (Kviklys et al., 2012). Significant rootstock behavior was established under varying growth conditions in a separate international trial, where the most productive trees were found on B.396 and M.9 rootstocks in Lithuania, on M.26 in Poland, on P 67 and P 22 in Latvia, and on M.26 in Estonia (Kviklys et al., 2013). Rootstock-scion or rootstock-location interactions were observed in NC-140 multi-location trials in the United States of America (Autio, 2001; Hirst et al., 2001), as well as in studies performed in other countries (Denardi et al., 2018).

Rootstock-cultivar interaction was found to be significant in evaluating the effect of rootstock on apple bearing stability (Kviklys et al., 2016). Alternate bearing is widespread in most of cultivated apple cultivars and causes serious economic losses for apple industry.

Plant vegetative and reproductive development is regulated by endogenous phytohormones. Phytohormones are translocated to the sites of action as signal molecules which affect tissue differentiation, and perform both above and below the grafted interface (Baron et al., 2019). Auxins and cytokinins (CKs) act antagonistically to regulate root and shoot growth, as well as the outgrowth of axillary meristems, while playing a role in each other’s synthesis and transport (Müller and Leyser, 2011). In contrast to auxins, CKs are produced in the root and translocated to the shoot, where they control important developmental processes such as shoot growth and productivity (Aloni et al., 2010). A decrease in the basipetal flow of indole-3 acetic acid (IAA) from the shoot stimulates the synthesis and export of CKs from the root. It was demonstrated that in contrast to non-grafted plants, grafting disturbed the auxin/CK balance (Sorce et al., 2002). Several studies have shown that, compared to vigorous rootstocks, dwarfing rootstocks (e.g., M.9) reduce the basipetal transport of IAA to the root, thereby reducing the amount of root-produced CK and gibberellin (GA) transported to the scion (Michalczuk, 2002; Van Hooijdonk et al., 2010), while others have shown an inverse relationship between the rate of IAA diffusion and the CK concentration in the xylem (Van Hooijdonk et al., 2011). Although GAs are well-known for promoting rosette flowering in herbaceous plants, they are commonly known to repress flowering in apple. A simple explanation for this repression is that GA induces the expression of *MdTFL1-1* which acts as a flowering repressor in apple (Peace et al., 2019). In addition, GAs are leading phytohormones that modulate apical meristem differentiation by downregulating their levels at the induction of dormancy, followed by upregulation during dormancy release or bud burst (Liu and Sherif, 2019). Abscisic acid (ABA) coordinates root and shoot growth in plants (Sharp and LeNoble, 2002), regulates tolerance responses to a number of stress factors, and is one of the main determinants responsible for triggering the dwarfing process in higher species (Tworkoski and Fazio, 2015). ABA homeostasis in plants is essential for normal growth and development processes, in which buds are both the target site for ABA activity, and the principal location for ABA metabolism and catabolism (Liu and Sherif, 2019). Multiple physiological and transcriptomic studies have indeed proposed a central role for ABA in the repression of bud activity during early apical meristem differentiation, whereby ABA would function as a signal in response to short autumn days and decreasing temperatures in order to induce dormancy (Li et al., 2018; Tylewicz et al., 2018; Liu and Sherif, 2019).

Dwarfing apple rootstocks produced higher ABA concentrations compared to vigorous apple rootstocks. While there is no consensus, it has been suggested that ABA may support the role of GA in vigorous rootstocks, as opposed to dwarfing rootstocks (Baron et al., 2019). The proposed role of GAs in the dwarfing response (Van Hooijdonk et al., 2010) has been unclear since Van Hooijdonk et al. (2011) reported that concentrations of GA_19_ were similar in the xylem sap collected throughout the growing season from scions grafted on to M.9 or MM.106 rootstocks, and suggested that GA_19_ may be a precursor of bioactive GA_1_, required for shoot extension growth. Lower (GA_19_) levels were found in root and xylem exudates of scion cultivars grafted on dwarfing rootstock, compared to those grafted on semi-vigorous rootstocks (Tworkoski and Fazio, 2016). Thus, the effects of hormone signaling may vary at different stages of establishing communication in the graft union. Else et al. (2018) suggested that the manner in which rootstocks imparted their control over grafted scions was complex and included a “filtering effect” of the graft union at significantly low flow rates, an augmentation of xylem sap constituents *via* passage through the union at increased flow rates, and an altered synthesis or metabolism of key rootstock-sourced hormone signals.

One of the main causes of biennial bearing in apple trees is the inhibition of flower initiation by adjacent developing fruits. The distance between flower clusters in 1 year has long been known to influence the development of floral buds in the following year (Nichols et al., 2011). This localized inhibition may depend on a critical ratio of inhibitor and promoter hormones that inhibits the flowering of apical meristems (Pellerin et al., 2012), or may be a result of carbon limitation and/or hormonal inhibition of floral initiation by nearby seeds (Dennis, 2000). Inhibition of floral initiation by endogenous GAs (possibly GA_1_, GA_4_, and iso-GA_7_) produced by seeds was confirmed in heavily cropped “Fuji” trees (Kittikorn et al., 2010) and “Golden Delicious” (Ramíırez et al., 2004). GAs and jasmonic acid (JA) act antagonistically, with JA detected at high concentrations in apple trees when GA concentrations are low, suggesting its key role in floral initiation (Kittikorn et al., 2010).

Numerous studies have demonstrated a clear connection between sugar-sensing pathways and phytohormone metabolism and signaling (Eveland and Jackson, 2012; Ljung et al., 2015; Foster et al., 2017). Hormonal signals may regulate rootstock-mediated vigor by modulating gene expression in the scion, including sink activity (Albacete et al., 2015). Invigorating rootstocks upregulate genes involved in carbohydrate metabolism and sugar transport in the scion apical meristem (Foster et al., 2017). Increased sink strength of the primary form of transported carbon in the shoot apex show the best correlation between sorbitol dehydrogenase and plant size in grafted apple trees (Jensen et al., 2010). In addition, sorbitol dehydrogenase activity is modulated by phytohormonal changes, and is negatively (by ABA) and positively (by CKs) correlated with drought stress-induced hormonal changes (Li and Li, 2007). Cheng et al. (2002) suggested that glucose increases ABA biosynthetic gene expression. Glucose and auxin act synergistically in plant development (Procko et al., 2014) and promote polar auxin transport (Hornitschek et al., 2012). Glucose and CKs also share several transcriptional targets. Moore et al. (2003) demonstrated that a mutant defective in glucose sensing was hypersensitive to CK and insensitive to auxin. There is also evidence of metabolite dynamics, especially of raffinose family oligosaccharides, being correlated with many cold-related gene expression changes during transition to flowering (Peace et al., 2019).

Although it is unclear whether the reduced sugar concentrations in dwarfing rootstocks are the cause or result of altered hormone levels or signaling, these metabolic changes have a significant effect on grafted apple tree growth and development. In order to identify the physiological processes and regulatory networks involved with rootstock-induced dwarfing, we compared the metabolic changes of phytohormones and soluble sugars in apical meristems from dwarfing and vigorous rootstocks. The aim of the present study was to evaluate interactions between apple rootstocks, scions and different crop load levels that could influence return bloom and identify the differences in physiological status between dwarfing and vigorous rootstocks.

## Materials and Methods

### Growing Conditions

Experiments investigating the effect of apple rootstock, scion cultivar, and crop load on apple return bloom were performed in Lithuania (55°60′ N, 23°48′ E) in 2014–2016. Apple cultivars “Ligol” (Poland) and ““Auksis” (Lithuania) on five rootstocks with contrasting induced vigor were tested: semi-dwarf M.26; dwarf M.9, B.396, and P 67; and super-dwarf P 22. The orchard was planted in 2005, at 4 m × 1.5 m intervals. Trees were trained as slender spindles. Pest and disease management was carried out according to the rules of integrated plant protection (Valiuškaitė et al., 2017).

Thinning of flower clusters and fruitlets was carried out before flowering at the pink bud stage and corrected after the June drop by adjusting three crop load levels: 75, 113, and 150 fruits per tree. Taking the average fruit weight into account, the expected yield at these crop levels was 21, 32, and 43 t ha^−1^ for “Auksis”, and 27, 41, and 54 t ha^−1^ for “Ligol”, respectively.

Return bloom was evaluated a year following crop load adjustment, at the balloon stage of flowering, by counting all the flower clusters per tree.

Bud development in September and November was defined according to Foster et al. (2003). Thirty buds from each grafting and crop load treatment were evaluated.

### Determination of Phytohormones

Approximately 0.5 g of fresh plant tissue (90–120 buds per treatment) was ground with liquid N_2_ and extracted with 5 ml of cold 50% (v/v) acetonitrile. An internal standard solution mixture containing isotope-labeled phytohormones (200 pmol IAA, 40 pmol GA_1_, GA_4_, and GA_7_, 100 pmol ABA, and 100 pmol zeatin) was added to the samples. Samples were purified using Chromabond HLB SPE cartridges (3 ml, 60 mg, Macherey-Nagel). Briefly, cartridges were conditioned with 2 ml MeOH and 2 ml H_2_O. Samples were applied to the cartridges, and the pass-through was collected. Sample elution was carried out with 30% acetonitrile, and each eluted sample was collected together with the pass-through. Samples were dried in a vacuum concentrator and dissolved in 50 µl of 30% (v/v) acetonitrile.

Phytohormone analysis was performed in buds collected in mid-September, according to the procedure described by Šimura et al. (2018) with modifications, using ultra performance liquid chromatography (UPLC; Waters) combined with mass spectrometry (LC-MS/MS). Separation of phytohormones (GAs: GA_1_, GA_4_, and GA_7_, IAA, JA, ABA, and zeatin) was performed using an Acquity BEH C18 column (1.0 × 150 mm; Waters). The mobile phase was A: water [with 0.02% (v/v) acetic acid], and B: acetonitrile with 0.02% (v/v) acetic acid, at a flow rate of 0.1 ml min^−1^. Gradient was maintained at 5% B for 3 min, raised to 40% B in 12 min, raised to 90% B in 1 min, maintained at 90% B for 1.5 min, and equilibrated at 5% B for 4 min, before starting the next injection. Hormones were detected with the Bruker Ultra HTC ion trap mass spectrometer in negative mode, using multiple reaction monitoring (MRM).

### Determination of Sugars

Approximately 0.5 g of fresh plant tissue (90–120 buds per treatment) was ground and diluted with deionized H_2_O. The extraction process was carried out for 4 h at 20°C with mixing. Samples were centrifuged at 14,000 × *g* for 15 min. A clean-up step was performed prior to the chromatographic analysis. Briefly, 1 ml of supernatant was mixed with 1 ml of 0.01% (w/v) ammonium acetate in acetonitrile, and incubated for 30 min at +4 °C. The samples were centrifuged at 14,000 × *g* for 15 min and filtered through a 0.22-µm PTFE syringe filter (VWR International, USA). The analyses were performed using a Shimadzu HPLC instrument (Japan) equipped with an evaporative light scattering detector (ELSD). The separation of fructose, glucose, and sucrose was performed using a Shodex VG-50 4D HPLC column with deionized water (mobile phase A) and acetonitrile (mobile phase B) gradient. The gradient was maintained at 88% B for 13 min, changed linearly to 70% B in 9 min, maintained at 70% B for 1 min, raised back to 88% B in 2 min, and the column was equilibrated to 88% B for 5 min. The flow rate was set at 0.8 ml min^−1^.

### Statistical Analysis

The experiment was carried out in a randomized complete block design with three replicates and one single tree in each plot. Data was processed using the XLStat software. Analysis of variance (ANOVA) was carried out along with the post-hoc Tukey’s HSD test for statistical analyses. Differences were considered to be significant at p < 0.05. Multivariate principal component analysis (PCA) was also performed. The agglomerative hierarchical cluster (AHC) analysis was used to generate similarity cluster diagrams based on the rootstock, crop load and metabolites similarity, and variables similarity.

## Results

Evaluation of the effect of rootstock on apple return bloom showed that the average highest number of flower clusters for both tested cultivars was recorded in trees on M.9 and P 67 rootstocks (**Table 1**). “Auksis” trees on M.9, and “Ligol” trees on P 67 and M.9 rootstocks produced a significantly higher number of flower clusters. A significantly lower return bloom was recorded for “Auksis” on B.396 rootstock, and for “Ligol” on P 22 rootstock.

**Table 1:** The effect of rootstock and crop load interaction on the return bloom of “Ligol” and “Auksis” apple trees (number of flower clusters tree^−1^).

Treatment	“Ligol”	“Auksis”	Average
**M.26 (75)**	153.0 a*	142.3 bc	147.7 a
**M.26 (113)**	34.7 f	117.3 cde	76.0 f
**M.26 (150)**	16.3 g	109.3 de	62.8 h
**M.9 (75)**	101.3 c	176.0 a	138.7 ab
**M.9 (113)**	125.3 b	120.7 cde	123.0 cd
**M.9 (150)**	23.7 fg	108.7 de	66.2 gh
**B.396 (75)**	92.7 cd	98.7 e	95.7 de
**B.396 (113)**	62.0 e	98.7 e	80.3 f
**B.396 (150)**	58.7 e	93.7 e	76.2 fg
**P 67 (75)**	130.0 b	131.0 bc	130.5 bc
**P 67 (113)**	82.0 d	124.3 cd	103.2 de
**P 67 (150)**	60.0 e	116.3 de	88.2 ef
**P 22 (75)**	86.3 cd	151.0 ab	118.7 cd
**P 22 (113)**	55.7 e	116.3 cde	86.0 ef
**P 22 (150)**	21.7 fg	102.7 de	62.2 h
**Effect of rootstock**			
**M.26**	68.0 b	123.0 b	95.5 b
**M.9**	83.4 a	135.1 a	109.3 a
**B.396**	71.1 b	97.0 c	84.1 c
**P 67**	90.7 a	123.9 b	107.3 a
**P 22**	54.6 c	123.3 b	88.9 bc
**Effect of crop load**			
**75**	112.7 a	139.8 a	126.2 a
**113**	71.9 b	115.5 b	93.7 b
**150**	36.1 c	106.1 c	71.1 c

*The different letters on the same column indicate significant differences between treatments, rootstock and crop load separately. The analysis was conducted with all data resulting from 9 trees of “Ligol” and “Auksis” apple trees grafted on M.26, M.9, B.396, P 67, and P 22, with crop-load adjusted to 75, 113, and 150 fruits per tree. The data were processed using analysis of variance (ANOVA), the Turkey (HSD) multiple range test at the confidence level p = 0.05.

Evaluation of the effect of crop load on apple return bloom showed that both cultivars responded in the same way, i.e., the higher the crop load established in the previous year, the lower the recorded return bloom. Significant differences were established among all crop load levels. However, suppression of return bloom was different for both cultivars when comparing the lowest and highest crop levels. The return bloom of “Ligol” at a crop load of 150 fruits per tree was lower by 68%, compared to that obtained at a crop load of 75 fruits per tree, while return bloom of “Auksis” was lower only by 24%.

Evaluation of the effect of rootstock and crop load interactions on cultivar response produced varying results. Return bloom of “Ligol” on all rootstocks significantly depended on crop load. Only crop load levels of 113 and 150 fruits per tree, established on B.396 rootstock, did not significantly differ. Differences between crop load effect on return bloom of “Auksis” were less pronounced. Significant differences were recorded by comparing only the highest and lowest crop load levels, with no differences found between adjacent crop load levels on almost all rootstocks, and no differences detected among all the levels on B.396 rootstock.

Generative bud development in September and November was defined for both varieties (**Table 2**). P 22 rootstock produced the slowest bud development for both varieties. In September, 85% and 75% of the buds developed until stage 4 (with formation of terminal floral meristems) in “Ligol” and “Auksis” apple trees respectively, while the remainder were at stage 5. Further development of terminal floral meristems with bractlets and sepals (stage 5) reached 75% and 100% in “Ligol” and “Auksis” apple trees, respectively, in November.

**Table 2:** Bud development (%) of “Ligol” and “Auksis” apple trees in autumn.

Rootstocks	“Ligol”			“Auksis”		
	September	November		September	November	
	Stage 4	Stage 5	Stage 5	Stage 4	Stage 5	Stage 5
**M.26**	85	15	75	75	25	100
**M.9**	85	15	75	75	25	90
**B.396**	85	15	75	75	25	100
**P 67**	85	15	75	75	25	100
**P 22**	60	40	75	50	50	75

Bud development established according to Foster et al. (2003). n = 90 buds per treatment.

**Figure 1:**
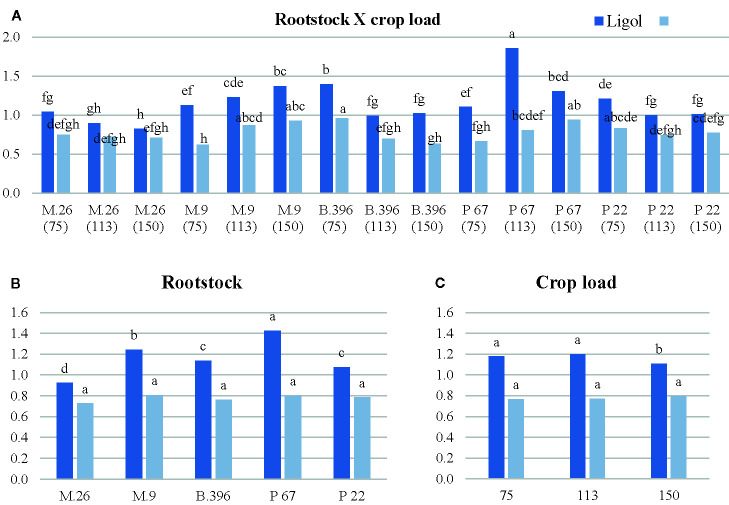
The effect of rootstock **(B)** and crop load **(C)** interaction **(A)** on the ratio of flowering promoters to inhibitors phytohormones in “Ligol” and “Auksis” buds. *The different letters on bars of same cultivar indicate significant differences. The analysis was conducted with all data resulting from 9 trees of “Ligol” and “Auksis” apple tree grafted on M.26, M.9, B.396, P 67, and P 22, with crop-load adjusted to 75, 113, and 150 fruits per tree. The data were processed using analysis of variance (ANOVA), the Turkey (HSD) multiple range test at the confidence level p = 0.05. Promoters—zeatin and jasmonic acid; Inhibitors—indolyl-3 acetic acid; abscisic acid; gibberellins (GA_7_, GA_3_, GA_1_).

**Figure 2:**
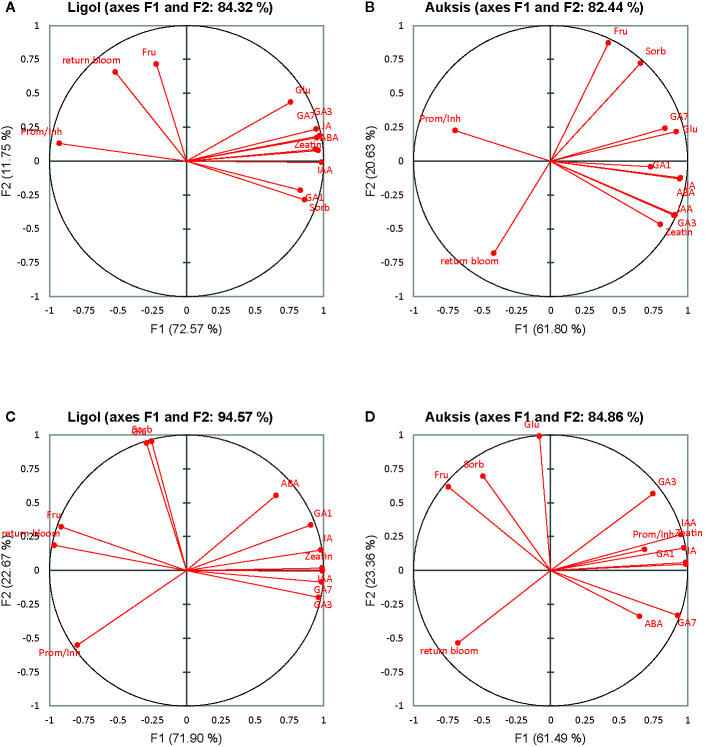
Correlation circle of phytohormones, soluble sugars and return bloom depending on rootstock **(A, C)** and on crop load **(B, D)** in “Ligol” and “Auksis” apple trees respectively. The analysis was conducted with all data resulting from 9 trees of “Ligol” and “Auksis” apple trees grafted on M.26, M.9, B.396, P 67, and P 22, with crop-load adjusted to 75, 113, and 150 fruits per tree. JA, jasmonic acid; IAA, indolyl-3 acetic acid; ABA, abscisic acid; GA_7_, GA_3_, GA_1_, gibberellins; Glu, glucose; Fru, fructose; Sorb, sorbitol.

Among flowering promoters, the phytohormone with the highest accumulation was JA, which ranged from 150 to 438 ng g^−1^ FW in both varieties. Among the inhibitor phytohormones the highest accumulation was observed in IAA and GA_7_. IAA concentrations ranged from 36 to 270 ng g^−1^ FW, and from 76 to 310 ng g^−1^ FW, while GA_7_ concentrations ranged from 36 to 187 ng g^−1^ FW, and from 96 to 354 ng g^−1^ FW in “Ligol” and “Auksis”, respectively (**Tables 2** and **3**). Compared to “Auksis”, such content range resulted in a higher ratio of promoter to inhibitor phytohormones in “Ligol” (**Figure 1A**). The significantly high content of all promoter and inhibitor phytohormones was observed in buds of semi-dwarfing M.26 rootstock with a crop load adjusted to 150 fruits per tree [M.26 (150)] in both varieties. Moreover, the accumulation of phytohormones and soluble sugars significantly increased with increasing crop load of “Auksis” on M.26, while an opposite tendency in soluble sugar accumulation in “Ligol” buds was observed (**Tables 3**–**5**). The lowest accumulation of all tested phytohormones was observed in buds of apple trees on dwarfing rootstocks M.9 and P 67, with crop load adjusted to 75 and 113, respectively in both varieties (**Tables 3** and **4**). The significant decrease in sugar contents in buds of “Ligol” was observed in the P 67 (crop load: 113 fruits per tree) treatment; however, M.9 (crop load: 150 fruits per tree) buds caused a significant decrease of sugars in “Auksis” (**Table 5**). Despite the content variation of phytohormones, the highest ratio of promoter to inhibitor phytohormones was in buds of “Ligol” on dwarfing P 67 rootstock with crop load adjusted to 113, while semi-dwarfing M.26 rootstock with crop load adjusted to 150 resulted in the lowest ratio of promoter to inhibitor phytohormones. However, a significant increase in the level of promoter to inhibitor hormones was observed in buds of “Auksis” on dwarfing rootstock B.396 (crop load: 75 fruits per tree) and a decrease was observed on M.9 (crop load: 75 fruits per tree) treatment (**Figure 1A**). Semi-dwarfing M.26 rootstock led to a significant increase, while dwarfing P 67 rootstock resulted in a significant decrease in phytohormone accumulation, in both varieties (**Tables 3** and **4**). The opposite effect of M.26 and P 67 rootstocks on the ratio of promoters to inhibitors was detected in “Ligol” trees (**Figure 1B**). However, neither rootstock nor crop load significantly affected the ratio of promoter to inhibitor phytohormones in “Auksis”. Although crop load did not significantly affect ABA accumulation, a load adjusted to 150 fruits per tree resulted in a significant increase in zeatin, JA, IAA, GA_7_, GA_3_, and GA_1_ levels in both varieties, in contrast to a load of 75 fruits per tree (**Tables 3** and **4**). No unique response was found for soluble sugar accumulation in apple tree buds (**Table 5**). M.26 rootstock produced a significant increase in glucose and sorbitol, while M.9 presented a significant increase in fructose in “Ligol” buds. A significant accumulation of all soluble sugars was detected in buds of “Auksis” on B.396 rootstock. A crop load of 75 fruits per tree significantly increased the content of glucose, fructose, and sorbitol in “Ligol”, while in “Auksis”, such increase was the result of crop load adjusted to 113 fruits per tree. Due to increased levels of inhibitor phytohormones, both rootstock and crop load produced either a strong or very strong negative correlation between the ratio of promoter to inhibitor phytohormones and analyzed phytohormones, while a positive correlation was found between the ratio of promoter to inhibitor phytohormones and fructose in “Ligol” (**Figures 2A, C**; **Table S1**). The correlation between the ratio of promoter to inhibitor phytohormones and sugars in buds of “Auksis” was not significant. In contrast to rootstock, crop load resulted in a strong correlation between the ratio of promoter to inhibitor phytohormones and zeatin, JA, IAA, ABA, GA_7_, GA_3_, and GA_1_ in “Auksis” (**Figures 2C, D**; **Table S2**).

**Table 3:** The effect of rootstock and crop load interaction on the accumulation of phytohormones in “Ligol” buds (ng g^−1^, FW).

Treatment	Promoters		Inhibitors				
	Zeatin	JA	IAA	ABA	GA_7_	GA_3_	GA_1_
**M.26 (75)**	9.04 c*	278.2 de	126.1 cde	8.59 d	108.7 e	18.8 e	12.8 e
**M.26 (113)**	10.94 b	353.1 b	198.1 b	11.02 b	148.1 c	30.2 b	17.2 c
**M.26 (150)**	12.83 a	428.0 a	270.1 a	13.45 a	187.4 a	41.5 a	21.6 ab
**M.9 (75)**	3.83 h	157.3 i	54.3 i	3.38 jkl	67.9 i	8.99 h	8.59 h
**M.9 (113)**	8.20 cd	336.2 b	105.5 fg	5.01 hi	129.2 d	26.9 c	13.9 de
**M.9 (150)**	6.64 ef	246.6 fg	84.3 h	2.82 l	77.2 h	10.2 gh	9.74 gh
**B.396 (75)**	6.56 ef	330.7 bc	104.9 fg	8.01 de	94.2 f	18.8 e	15.8 cd
**B.396 (113)**	5.51 fg	261.5 ef	110.8 efg	7.12 ef	112.3 e	21.6 d	16.6 c
**B.396 (150)**	8.77 c	351.8 b	135.8 cd	9.78 c	164.4 b	19.1 e	22.5 a
**P 67 (75)**	5.08 g	179.2 i	48.4 ij	5.49 gh	86.0 g	15.1 f	11.9 efg
**P 67 (113)**	3.72 h	161.8 i	35.8 j	3.20 kl	36.0 j	4.96 i	8.95 h
**P 67 (150)**	7.04 e	302.6 cd	119.9 def	3.50 jkl	85.2 gh	18.4 e	10.4 fgh
**P 22 (75)**	7.38 de	330.6 bc	137.8 c	6.22 fg	89.1 fg	26.2 c	19.5 b
**P 22 (113)**	7.20 de	222.7 gh	113.9 efg	4.34 ij	88.3 fg	11.2 gh	12.4 ef
**P 22 (150)**	7.19 de	212.4 h	101.5 g	4.17 ijk	88.3 fg	11.6 g	11.8 efg
**Effect of rootstock**							
**M.26**	10.94 a	353.1 a	198.1 a	11.02 a	148.1 a	30.2 a	17.2 b
**M.9**	6.22 c	246.7 c	81.4 c	3.74 d	91.4 c	15.4 c	10.7 d
**B.396**	6.95 b	314.7 b	117.2 b	8.30 b	123.7 b	19.8 b	18.3 a
**P 67**	5.28 d	214.5 d	68.0 d	4.06 d	69.1 d	12.8 d	10.4 d
**P 22**	7.26 b	255.2 c	117.7 b	4.91 c	88.6 c	16.3 c	14.6 c
**Effect of crop load**							
**75**	6.38 c	255.2 c	94.3 c	6.34 a	89.2 c	17.6 c	13.7 b
**113**	7.11 b	267.0 b	112.8 b	6.14 a	102.8 b	19.1 b	13.8 b
**150**	8.49 a	308.3 a	142.3 a	6.74 a	120.5 a	20.1 a	15.2 a

*The different letters on the same column indicate significant differences between treatments, rootstock and crop load separately. The analysis was conducted with all data resulting from 9 trees of “Ligol” apple tree grafted on M.26, M.9, B.396, P 67, and P 22, with crop-load adjusted to 75, 113, and 150 fruits per tree. The data were processed using analysis of variance (ANOVA), the Turkey (HSD) multiple range test at the confidence level p = 0.05.

JA, jasmonic acid; IAA, indolyl-3 acetic acid; ABA, abscisic acid; GA_7_, GA_3_, GA_1_, gibberellins.

**Table 4:** The effect of rootstock and crop load interaction on the accumulation of phytohormones in “Auksis” buds (ng g^−1^. FW).

Treatment	Promoters		Inhibitors				
	Zeatin	JA	IAA	ABA	GA_7_	GA_3_	GA_1_
**M.26 (75)**	9.04 c*	278.2 cd	166.1 cde	8.59 de	188.7 d	18.8 g	2.78 gh
**M.26 (113)**	10.9 b	358.1 b	238.1 b	11.0 b	213.1 c	35.2 b	12.2 cd
**M.26 (150)**	12.8 a	438.0 a	310.1 a	13.5 a	237.4 b	51.5 a	21.6 a
**M.9 (75)**	3.83 hi	157.3 e	94.3 i	3.38 hi	147.9 f	8.99 i	4.59 efg
**M.9 (113)**	8.20 cd	326.2 bc	148.8 efg	5.01 gh	189.2 d	36.9 b	3.87 fgh
**M.9 (150)**	6.64 ef	256.6 d	124.3 h	2.82 i	127.2 g	20.2 g	9.74 d
**B.396 (75)**	6.56 ef	330.7 b	144.9 fg	8.01 de	174.2 e	18.8 g	5.83 ef
**B.396 (113)**	5.51 fg	251.5 d	150.8 efg	7.12 ef	172.3 e	31.6 c	6.60 e
**B.396 (150)**	8.77 c	361.8 b	172.4 cd	9.78 bcd	354.4 a	29.1 d	22.5 a
**P 67 (75)**	5.08 gh	179.2 e	88.4 ij	5.49 fg	166.0 e	15.1 h	1.86 h
**P 67 (113)**	3.72 i	151.8 e	75.8 j	3.20 hi	96.0 h	15.0 h	2.95 gh
**P 67 (150)**	7.04 de	312.6 bc	163.2 cdef	3.50 ghi	135.3 fg	28.4 de	10.4 d
**P 22 (75)**	6.06 efg	327.3 bc	176.8 c	10.8 bc	167.3 e	32.3 c	14.8 bc
**P 22 (113)**	5.54 fg	247.6 d	156.5 def	7.37 ef	136.1 fg	24.6 f	15.5 b
**P 22 (150)**	6.31 efg	239.8 d	133.8 gh	8.97 cde	132.1 g	26.2 ef	15.7 b
**Effect of rootstock**							
**M.26**	10.9 a	358.1 a	238.1 a	11.0 a	213.1 b	35.2 a	12.2 b
**M.9**	6.22 c	246. 7 c	122.5 c	3.74 d	154.8 c	22.02 d	6.07 c
**B.396**	6.95 b	314.7 b	156.0 b	8.30 c	233.7 a	26.5 c	11.7 b
**P 67**	5.28 d	214.5 d	109.1 d	4.06 d	132.4 e	19.5 e	5.07 c
**P 22**	5.97 c	271.6 c	155.7 b	9.05 b	145.1 d	27.7 b	15.3 a
**Effect of crop load**							
**75**	6.12c	254.5 b	134.1 c	7.25 ab	168.8 b	18.8 c	5.97 c
**113**	6.78 b	267.0 b	154.0 b	6.74 b	161.4 c	28.7 b	8.22 b
**150**	8.32 a	321.8 a	180.8 a	7.70 a	197.3 a	31.1 a	16.0 a

*The different letters on the same column indicate significant differences between treatments, rootstock and crop load separately. The analysis was conducted with all data resulting from 9 trees of “Auksis” apple tree grafted on M.26, M.9, B.396, P 67, and P 22, with crop-load adjusted to 75, 113, and 150 fruits per tree. The data were processed using analysis of variance (ANOVA), the Turkey (HSD) multiple range test at the confidence level p = 0.05.

JA, jasmonic acid; IAA, indolyl-3 acetic acid; ABA, abscisic acid; GA_7_, GA_3_, GA_1_, gibberellins.

**Table 5:** The effect of rootstock and crop load interaction on the accumulation of soluble sugars in “Ligol” and “Auksis” buds (mg g^−1^. FW).

Treatment	“Ligol”			“Auksis”		
	Glu	Fru	Sorb	Glu	Fru	Sorb
**M.26 (75)**	2.02 a*	0.21 ab	0.42 a	0.97 bcde	0.10 cd	1.76 cde
**M.26 (113)**	1.02 bc	0.10 de	0.18 bc	1.08 bcde	0.12 bcd	1.99 bcde
**M.26 (150)**	0.59 cde	0.06 e	0.19 bc	2.31 a	0.13 bc	2.39 abcd
**M.9 (75)**	0.99 bc	0.22 a	0.21 b	0.39 de	0.08 cd	1.25 ef
**M.9 (113)**	0.38 de	0.18 abcd	0.08 d	0.90 bcde	0.12 bcd	1.68 def
**M.9 (150)**	1.23 b	0.28 a	0.27 b	0.28 e	0.05 d	0.72 f
**B.396 (75)**	0.71 cd	0.18 abcd	0.22 b	1.32 bc	0.19 ab	2.70 abc
**B.396 (113)**	0.57 cde	0.22 a	0.21 b	1.64 ab	0.21 a	3.16 a
**B.396 (150)**	0.89 bc	0.05 e	0.19 b	1.23 bcd	0.19 ab	3.24 a
**P 67 (75)**	0.88 bc	0.20 abc	0.23 b	1.17 bcde	0.22 a	3.05 a
**P 67 (113)**	0.22 e	0.07 e	0.09 cd	0.56 cde	0.11 bcd	1.20 ef
**P 67 (150)**	0.84 bcd	0.05 e	0.22 b	0.45 cde	0.08 cd	1.19 ef
**P 22 (75)**	0.65 cde	0.10 de	0.25 b	0.40 de	0.09 cd	1.18 ef
**P 22 (113)**	0.75 cd	0.12 bcde	0.21 b	2.23 a	0.18 ab	2.94 ab
**P 22 (150)**	0.70 cd	0.11 cde	0.23 b	0.58 cde	0.10 cd	1.69 de
**Effect of rootstock**						
**M.26**	1.21 a	0.12 b	0.26 a	1.45 a	0.12 bc	2.05 b
**M.9**	0.87 b	0.23 a	0.18 bc	0.52 c	0.08 c	1.22 c
**B.396**	0.72 b	0.15 b	0.21 bc	1.40 a	0.20 a	3.03 a
**P 67**	0.64 b	0.11 b	0.18 c	0.73 bc	0.14 b	1.81 b
**P 22**	0.70 b	0.11 b	0.23 ab	1.07 ab	0.12 b	1.94 b
**Effect of crop load**						
**75**	1.05 a	0.18 a	0.26 a	0.85 b	0.14 ab	1.99 a
**113**	0.59 c	0.14 b	0.15 c	1.28 a	0.15 a	2.19 a
**150**	0.85 b	0.11 b	0.22 b	0.97 ab	0.11 b	1.86 a

*The different letters on the same column indicate significant differences between treatments, rootstock and crop load separately. The analysis was conducted with all data resulting from 9 trees of “Ligol” and “Auksis” apple trees grafted on M.26, M.9, B.396, P 67, and P 22, with crop-load adjusted to 75, 113, and 150 fruits per tree. The data were processed analysis of variance (ANOVA), the Turkey (HSD) multiple range test at the confidence level p = 0.05.

Glu, glucose; Fru, fructose; Sorb, sorbitol.

**Figure 3:**
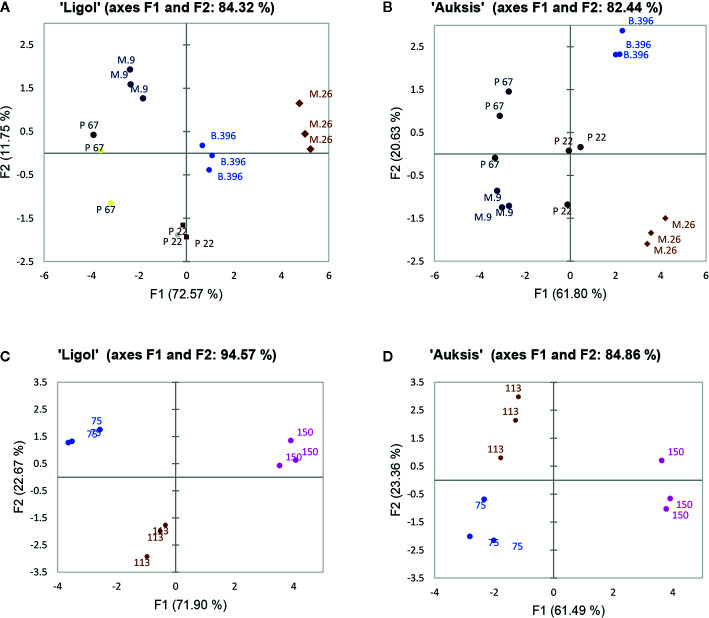
The PCA scatterplot, indicating distinct differences in phytohormones, soluble sugars and return bloom depending on rootstock **(A, C)** and crop load **(B, D)** in “Ligol” and “Auksis” apple trees respectively. The analysis was conducted with all data resulting from 9 trees of “Ligol” and “Auksis” apple trees grafted on M.26, M.9, B.396, P 67, and P 22, with crop-load adjusted to 75, 113, and 150 fruits per tree.

PCA results show the average coordinates of individual phytohormones (zeatin, JA, IAA, ABA, GA_7_, GA_3_, and GA_1_), soluble sugars (glucose, fructose, and sorbitol), ratio of promoter to inhibitor phytohormones, and return bloom, in response to rootstocks with different growth vigor, and crop load. The first two factors (F1 vs. F2) of the PCA, as shown in the correlation circle (**Figure 2**) and scatterplot (**Figure 3**), explained 84.32% and 82.44% of the total data variance of rootstocks (**Figures 3A, C**), as well as 94.57% and 84.86% of that of crop load (**Figures 3B, D**) in “Ligol” and “Auksis”, respectively. F1 explained 11%–23% of the total variance, whereas F2 explained 72.57% and 61.80% (effect of rootstock on “Ligol” and “Auksis”, respectively), as well as 71.90% and 61.49% (effect of crop load for “Ligol” and “Auksis”, respectively) of the total variability. Therefore, F2 described the disparity among rootstock and crop load effects. To summarize all the effects observed in the PCA scatter plot, the effect of the semi-dwarfing M.26 rootstock was significantly different from that of the dwarfing M.9 and P 67 rootstocks, in both varieties (**Figures 3A, C**). The super-dwarfing P 22 rootstock also presented a similar metabolic response of phytohormones and sugars to dwarfing rootstocks. The most intensive crop load (150 fruits per apple tree) produced a significantly different response to less intensive crop loads (113 and 75) in both varieties (**Figures 3B, D**).

**Figure 4:**
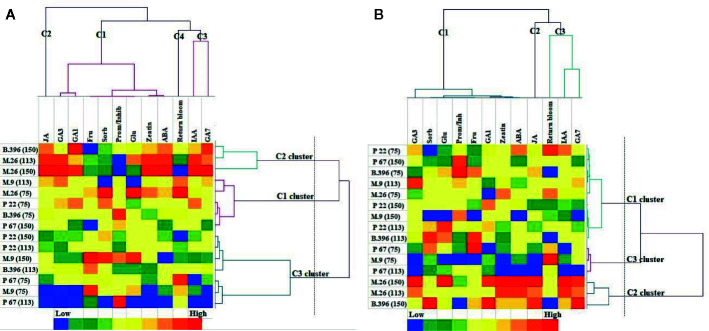
Agglomerative hierarchical cluster (AHC) analysis in “Ligol” **(A)** and “Auksis” **(B)**. Phytohormones, soluble sugars and return bloom grouped by similarities in concentration mean and intensity values. The analysis was conducted with all data resulting from 9 trees of “Ligol” and “Auksis” apple trees grafted on M.26, M.9, B.396, P 67, and P 22, with crop-load adjusted to 75, 113, and 150 fruits per tree.

The AHC analysis was used to divide the rootstock and crop load into groups of increasing dissimilarity. Three clusters were identified in both “Ligol” (**Figure 4A**) and “Auksis” (**Figure 4B**) samples. This division correspond to apple rootstocks grouped according to vigor and crop load in cluster 2 [M.26(113), M.26(150), B.396(150)] and cluster 3 [M.9(75), P 67(75), P 67(113)], but did not correspond in cluster 1 [M.26(75), M.9(113), B.396(75), P 67(150), P 22(75)] in both varieties. Rootstocks M.9 and P 22 with crop load of 150 fruits per tree and rootstocks B.396 and P 22 with crop load of 113 fruits per tree belonged to cluster 3 in “Ligol” and to cluster 1 in “Auksis”. With regards to bud phytohormones and sugars concentrations and return bloom four clusters were identified in “Ligol” (**Figure 4A**) and three clusters were identified in “Auksis” (**Figure 4B**). Group, which included M.9 and P 67 rootstocks with crop load of 75 fruits per tree and P 67 with crop load of 113 fruits per tree, was characterized by low JA (cluster 2), GA_7_, and IAA (cluster 3) concentrations in both varieties. Other rootstock and crop load combinations distinguished in medium to high JA, GA_7_, and IAA concentrations. The return bloom was in the same cluster with IAA and GA_7_ (C3) in “Auksis” (**Figure 4B**), while in “Ligol” return bloom was in C4, but IAA and GA_7_ in C3 clusters (**Figure 4A**). Lower contents of inhibitory phytohormones (IAA and GA_7_) resulted in higher values of return bloom and vice versa. Other tested phytohormones and sugars were in C1 in both varieties. In contrast to “Auksis”, during generative bud development in September (**Table 1**), semi dwarfing M.26 rootstock with crop load of 113 and 150 fruits per tree (cluster 2) distinguished in lower soluble sugars contents and low ratio of promoter to inhibitor phytohormones (**Figure 4**). Super dwarfing rootstock P 22 (cluster 1 and 3 for “Ligol”, cluster 1 for “Auksis”) showed low to medium contents of tested metabolites. There was no common tendency established for metabolite variation in dwarfing rootstocks M.9, B.396, and P 67.

## Discussion

Fruit tree species from the Rosaceae family are non-sensitive to changes in photoperiod and required mainly low nighttime temperatures to become floral (Hanninen and Tanino, 2011). Fadón et al. (2020) suggested that winter dormancy induction is associated with leaf fall and refers to growth suppression imposed on particular organs by other tree structures (e.g., apical dominance) due to the production and/or action of inhibitory molecules. Hoover et al. (2004) and Mcartney et al. (2001) noticed that the duration of doming differs in different apple tree cultivars and the timing of floral commitment is not related either to the time of flowering, or to the time of fruit maturity of the cultivar the other cultivars. We found that apical meristems formed terminal floral meristems in both “Ligol” and “Auksis” cultivars (**Table 2**) in September. Therefore, with regards to morphological changes inside the buds, they became florally committed at the end of the growing season. Foster et al. (2003) found that the gynoecium and stamens were not distinguishable in *Malus* spp. during dormancy initiation, suspending flower development during early stages of apical meristems differentiation. Phytohormones such as ABA, GAs, IAA, CK, and possibly JA were implicated in direct or indirect regulation of different phase transitions during floral initiation processes (Charrier et al., 2011; Liu and Sherif, 2019) for the following year (Stephan et al., 2001). Compared to analyzed phytohormones, apple buds distinguished in the highest accumulation of JA, followed by GA_7_, and IAA in September (**Tables 3** and **4**). Moreover, a strong positive correlation between JA and inhibitor phytohormones was found (**Figure 2**; **Tables S1** and **S2**), approved by AHC analysis (**Figure 4**) suggesting the important role of JA in apple bud developmental processes. ABA, a growth inhibitor and storage promoter, has been described as a key hormonal regulator in the floral initiation processes. In many plant species, ABA is antagonized by the growth promoter GA, which act as floral inhibitors (Wilkie et al., 2008; Fadón et al., 2020). However, in concurrence with Peace et al. (2019), ABA and GAs in apple trees acted synergistically, a positive correlation was found between ABA and GAs in both varieties (**Figure 2**; **Tables S1** and **S2**; **Figure 4**). At the early stage of apical meristem differentiation, GAs may activate the metabolic pathways, even though a decline in GA (especially GA_3_ and GA_4_) levels induces growth cessation and bud set. GA_4_ treatment led to the enhancement of several energy metabolism pathways, including those associated with sugar metabolism (Zhuang et al., 2015). In contrast to rootstock, crop load resulted in negative correlations between GAs (GA_1_, GA_3_, and GA_7_) and soluble sugars (glucose and sorbitol) (**Figure 2**; **Tables S1** and **S2**). Nonstructural carbohydrate dynamics have often been assigned as long-distance sugar signaling pathway in developmental changes in shoot apical meristems (Moghaddam and Van den Ende, 2013). Fadón et al. (2020) stated that trees exhibit strong fluctuations in early shoot apical meristem developmental stages in the rates at which soluble sugars (i.e., glucose, fructose, and sorbitol) and starch are synthesized and degraded. In late autumn and during winter, carbohydrate synthesis declines progressively until leaf fall, with nonstructural carbohydrates acting as reserve molecules, and possibly supporting future growth. Xylem transport is progressively limited (by IAA and CK antagonistic action in the shoot apical meristem) in autumn, because leaf senescence and leaf fall cause a progressive reduction in transpiration (Aloni et al., 2010; Müller and Leyser, 2011). Li et al. (2012) indicated that apple is unique in terms of sugar metabolism and accumulation, as almost all sorbitol and a half of sucrose are converted to hexoses by invertase.

Aloni et al. (2010) suggested that invigorating properties of the rootstocks induced a higher growth rate in the scion, possibly by increasing the supply of CK to the shoot and decreasing that of IAA. In addition, rootstocks with dwarfing characteristics similar to those of M.9 and MM.106 indicated that altered auxin transport was also likely to be involved, with dwarfing rootstocks exhibiting a reduced capacity for polar auxin transport (Else et al., 2018). Compared to dwarfing rootstocks, the semi-dwarfing M.26 rootstock induced the accumulation of zeatin and IAA in buds of both apple tree varieties (**Tables 3** and **4**). Baron et al. (2019) found that dwarfing apple rootstocks contained lower amounts of plant growth promoter phytohormones, but higher amounts of inhibitor phytohormones, than vigorous rootstocks of the same species, with high ABA levels present in dwarfing rootstock stems. We found that buds of “Ligol” on dwarfing rootstocks accumulated larger amounts of flowering promoter phytohormones (zeatin and JA) compared to the semi-dwarfing M.26 rootstock (**Figure 1B**), and that buds of dwarfing rootstocks presented lower ABA levels in both varieties (**Tables 3** and **4**). Though dwarfing rootstocks of apple plants contain large amounts of ABA in their xylem (Lordan et al., 2017; Else et al., 2018), ABA accumulation in buds was induced by the more vigorous M.26 rootstock (**Tables 3** and **4**). While ABA can limit extension growth by suppressing GA_1_ accumulation (Benschop et al., 2005), it is not known whether rootstock-sourced ABA and scion-derived GAs interact to regulate shoot extension in grafted scions. Van Hooijdonk et al. (2010) determined that exogenously applied GA_4+7_ primarily reduced the proportion of primary and secondary shoots that presented early terminated growth, thereby increasing the final length and node number of “Royal Gala” shoots on M.9. An endogenous signaling mechanism is proposed here, whereby dwarfing rootstocks reduce the basipetal transport of IAA to the root, consequently decreasing the amount of root-produced CK and GA transported to the scion. However, no evidence was found to suggest that the dwarfing capacity of M.9 rootstocks could be attributed to a reduced supply of CKs from the rootstock to the scion (Else et al., 2018). Compared to the semi-dwarfing M.26 rootstock, dwarfing rootstocks (M.9, B.396, P 67, and P 22) resulted in a decrease of zeatin, JA, IAA ABA, and GAs except for an increase of GA_1_ in “Ligol” and an increase of GA_7_ in “Auksis” on B.396 (**Tables 2** and **3**). This could explain why the return bloom on B.396 rootstock was markedly the lowest for “Auksis” and average for both cultivars (**Table 1**). However, it should be noted that the accumulation, and especially the ratio, of these phytohormones was highly dependent on the developmental stage of apical meristems.

IAA and ABA can act as control factors in the ripening process, as sharp increases in the accumulation of both hormones occurred, and were followed by, the corresponding fruit quality indices. A significant increase of IAA concentration in the leaves of “Ligol” trees grafted on M.9 rootstock lead to slower ripening, while a significant increase of ABA concentration, together with the highest ripening rate, were detected in “Ligol” apple trees with the lowest crop load (Samuolienė et al., 2019).

Foster et al. (2017) found that compared to the dwarfing M.27 and the vigorous M.793 rootstocks, fructose and glucose concentrations were significantly lower in stems (above and below the graft junction) and roots tissues of the “Royal Gala” M.9 trees. This suggested that the low concentration of glucose, fructose, and myo-inositol in trees with M.9 rootstock would have a significant effect on the physiology of both rootstock and scion. In comparison to semi-dwarfing or super-dwarfing rootstocks, a similar decrease in soluble sugars in “Ligol” leaves from dwarfing P 67 and B.396 rootstocks was reported by Samuolienė et al. (2016). In agreement with previously obtained data, the significant decrease in fructose, glucose, and sorbitol levels in “Ligol” buds was also found in less vigorous rootstocks, except for a significantly higher fructose concentration in buds of “Ligol” trees on M.9 rootstock. A significant increase in soluble sugar content in buds of “Auksis” on dwarfing B.396 rootstock was observed, while no difference in glucose accumulation was observed between the semi-dwarfing M.26, dwarfing B.396, and super-dwarfing P 22 rootstocks (**Table 5**). The differences in soluble sugar accumulation in buds produced by these rootstocks may be related to the different periods of time required for fruit ripening between “Ligol” and “Auksis”. Guitton et al. (2016) suggested that competition for carbohydrates between the developing fruit and nearby apical buds lead to local carbon depletion and reduced cellular activity in the vegetative meristems, thus blocking the onset of floral development. While all sugars are transported *via* the sap to the buds just before budburst, carbohydrate dynamics are restricted to the bud tissues during early stages of apical meristems differentiation. Therefore, soluble sugar levels were increased in (Signorelli et al., 2018). Rootstock-dependent positive correlations between glucose and IAA, as well as between glucose and ABA (**Figure 2**; **Tables S1** and **S2**) indicated that glucose, IAA, and ABA act synergistically and that apple tree flower induction was influenced by sugar metabolism, as well as auxin and ABA signaling pathways. However, crop load-dependent correlations between glucose, sorbitol, and phytohormones were not significant in both varieties.

Tromp (2000), in his review on the effect of thinning on return bloom, indicated that the flower formation process is not sufficiently understood, especially in the early phases. Return bloom generally decreases as crop load increases (Serra et al., 2016). Marini et al. (2013) found that flower density was negatively correlated with the previous season’s crop load and that the interaction between rootstock and crop load was not established. Our data confirmed a negative correlation between crop load and return bloom (**Table 1**) of both tested cultivars, based on the averaged data obtained from all rootstocks. The highest crop load significantly suppressed return bloom and supported the findings of Embree et al. (2007), where biennial bearing was observed in “Honeycrisp” apple trees thinned to 150 blossom clusters. However, the effect of rootstock on the suppression of return bloom when comparing the lowest and highest crop levels was different for both cultivars. “Ligol” on M.26 rootstock recorded a decrease in flowering of approximately 90%, while a decrease of approximately 75% was recorded on M.9 and P 22 rootstocks. Meanwhile, the highest decrease in flowering (38%) was recorded for “Auksis” on M.9 rootstock. A very stable return bloom of “Auksis” was produced on B.396 and P 67 rootstocks, where differences between contrasting levels were only 5% and 11% respectively. This, however, cannot be explained by the ratio of flowering promoters to inhibitor phytohormones (**Figure 1**), indicating that bearing stability is more dependent on these rootstocks, but not on the crop load. Rootstock-scion interactions on return bloom were detected in our previous study (Kviklys et al., 2016) and in the study of 48 apple rootstocks, where several Geneva rootstocks were distinguished by low alternate bearing (Reig et al., 2018).

Return bloom of “Ligol” significantly correlated with the ratio of promoter and inhibitor hormones, while no such correlation was established for “Auksis” (**Tables S1 **and **S2**). The AHC analysis showed that return bloom was more dependent on IAA, GA_7_ and JA, rather than on other phytohormones or sugars in both varieties. Generally, higher correlations between phytohormones, soluble sugars, and return bloom were established, evaluating the effect of crop load, but not that of rootstocks. Return bloom of both cultivars on all rootstocks negatively correlated with the amount of sorbitol in buds, and additionally with the amount of fructose in the case of “Auksis”.

The PCA scatter plot on the metabolic response of phytohormones and sugars indicated that the effect of the semi-dwarfing M.26 rootstock was significantly different from that of the dwarfing M.9 and P 67, as well as of the super-dwarfing P 22 rootstocks in both varieties. The most intensive crop load (150 fruits per apple tree) produced a significantly different response compared to less intensive crop loads (113 and 75) in both varieties. In contrast to soluble sugar accumulation, increased crop load resulted in increased levels of phytohormones, but did not affect ABA accumulation.

Dwarfing rootstocks M.9, B.396, and P 67, as well as the super-dwarf P 22 resulted in altered accumulation of promoter phytohormones, while the more vigorous semi-dwarfing M.26 rootstock induced a higher content of glucose in “Ligol” buds, of glucose and sorbitol in “Auksis” buds, and of inhibitory phytohormones, by increasing the levels of IAA, ABA, and GAs. Therefore, soluble sugars, especially glucose, and hormonal pathways were interconnected and acted together to control flower induction, while being both rootstock- and crop load-dependent.

Apple return bloom was dependent both on rootstock and crop load. Interactions between rootstocks and cultivars were established: M.9 rootstock determined the highest return bloom for “Auksis”, and P 67 rootstock for “Ligol”. The lowest return bloom was recorded on the super dwarfing P 22 rootstock in “Ligol”, and on the dwarfing B.396 in “Auksis”. Comparing the effect of rootstock on return bloom under different crop load levels showed that the most significant decrease in return bloom resulted from the highest crop load in “Auksis” grafted on M.9 and P 22 rootstocks, and in “Ligol” grafted on P 22 rootstock.

## Data Availability Statement

All datasets presented in this study are included in the article/**Supplementary Material**.

## Author Contributions

DK—trial setup, field trials, data analysis, and writing of the manuscript. GS—chromatographic analysis, data analysis, and writing of the manuscript. All authors contributed to the article and approved the submitted version.

## Funding

This research was funded by a grant (No. MIP-036-2014) from the Research Council of Lithuania.

## Conflict of Interest

The authors declare that the research was conducted in the absence of any commercial or financial relationships that could be construed as a potential conflict of interest.
