# Supplementary material for: Relationships Among the Rootstock, Crop Load, and Sugar Hormone Signaling of Apple Tree, and Their Effects on Biennial Bearing
Source: Front Plant Sci. 2020 Aug 7;11:1213. doi: 10.3389/fpls.2020.01213 (PMC7427310; doi:10.3389/fpls.2020.01213)
Supplement: Supplementary file 1 [file Table_1.docx]

**SUPPLEMENTAL DATA**

**TABLE S1** The correlation matrix (Pearson (n)) between phytohormones, soluble sugars and return bloom in cv. ʹLigolʹ apple tree.

|  |  | **Crop load** | | | | | | | | | | |  |
| --- | --- | --- | --- | --- | --- | --- | --- | --- | --- | --- | --- | --- | --- |
|  | Variables | Zeatin | JA | IAA | ABA | GA_7_ | GA_3_ | GA_1_ | Prom/Inh | Glu | Fru | Sorb | Return bloom |
| **Rootstocks** | Zeatin | **1** | **0.98** | **0.99** | **0.63** | **0.99** | **0.94** | **0.91** | **-0.80** | **-0.27** | **-0.89** | **-0.24** | **-0.94** |
|  | JA | **0.87** | **1** | **0.97** | **0.68** | **0.96** | **0.91** | **0.96** | **-0.86** | **-0.14** | **-0.84** | **-0.09** | **-0.92** |
|  | IAA | **0.98** | **0.91** | **1** | **0.61** | **0.99** | **0.94** | **0.90** | **-0.81** | **-0.30** | **-0.89** | **-0.25** | **-0.97** |
|  | ABA | **0.87** | **0.96** | **0.91** | **1** | **0.53** | **0.56** | **0.75** | **-0.74** | **0.31** | **-0.49** | **0.31** | **-0.48** |
|  | GA_7_ | **0.88** | **0.99** | **0.90** | **0.96** | **1** | **0.95** | **0.88** | **-0.75** | **-0.36** | **-0.91** | **-0.33** | **-0.97** |
|  | GA_3_ | **0.97** | **0.94** | **0.97** | **0.95** | **0.95** | **1** | **0.80** | **-0.63** | **-0.47** | **-0.97** | **-0.43** | **-0.96** |
|  | GA_1_ | **0.66** | **0.88** | **0.75** | **0.85** | **0.84** | **0.73** | **1** | **-0.88** | 0.02 | **-0.67** | **0.10** | **-0.80** |
|  | Prom/Inh | **-0.90** | **-0.83** | **-0.92** | **-0.78** | **-0.83** | **-0.85** | **-0.77** | **1** | **-0.30** | **0.54** | **-0.33** | **0.70** |
|  | Glu | **0.83** | **0.70** | **0.77** | **0.67** | **0.74** | **0.83** | **0.35** | **-0.65** | **1** | **0.54** | **0.96** | **0.45** |
|  | Fru | **-0.20** | **-0.07** | **-0.27** | **-0.25** | -0.01 | **-0.15** | **-0.28** | **0.13** | **0.03** | **1** | **0.56** | **0.94** |
|  | Sorb | **0.84** | **0.72** | **0.87** | **0.71** | **0.69** | **0.79** | **0.65** | **-0.84** | **0.63** | **-0.39** | **1** | **0.41** |
|  | Return bloom | **-0.44** | **-0.35** | **-0.49** | **-0.33** | **-0.34** | **-0.34** | **-0.55** | **0.71** | **-0.11** | **0.26** | **-0.64** | **1** |

Values in bold are different from 0 with a significance level alpha=0.95

**TABLE S2** The correlation matrix (Pearson (n)) between phytohormones, soluble sugars and return bloom in cv. ʹAuksisʹ apple tree.

|  | |  | **Crop load** | | | | | | | | | | | | | | | | | | | | |  |  |
| --- | --- | --- | --- | --- | --- | --- | --- | --- | --- | --- | --- | --- | --- | --- | --- | --- | --- | --- | --- | --- | --- | --- | --- | --- | --- |
|  | | Variables | Zeatin | | JA | | IAA | | ABA | | GA_7_ | | GA_3_ | | GA_1_ | | Prom/Inh | | Glu | | Fru | Sorb | | Return bloom |  |
| **Rootstocks** | | Zeatin | | **1** | **0.96** | | **0.97** | | **0.59** | | **0.86** | | **0.84** | | **0.98** | | **0.62** | | **0.09** | | **-0.62** | | **-0.35** | **-0.73** | |
|  |  | JA | | **0.88** | **1** | | **0.95** | | **0.63** | | **0.90** | | **0.75** | | **0.98** | | **0.76** | | -0.02 | | **-0.70** | | **-0.40** | **-0.65** | |
|  |  | IAA | | **0.95** | **0.92** | | **1** | | **0.45** | | **0.80** | | **0.90** | | **0.97** | | **0.63** | | **0.19** | | **-0.58** | | **-0.31** | **-0.77** | |
|  |  | ABA | | **0.73** | **0.88** | | **0.90** | | **1** | | **0.74** | | **0.13** | | **0.58** | | **0.58** | | **-0.38** | | **-0.56** | | **-0.32** | **-0.25** | |
|  |  | GA_7_ | | **0.66** | **0.84** | | **0.64** | | **0.62** | | **1** | | **0.48** | | **0.91** | | **0.56** | | **-0.39** | | **-0.88** | | **-0.66** | **-0.43** | |
|  |  | GA_3_ | | **0.89** | **0.91** | | **0.98** | | **0.94** | | **0.63** | | **1** | | **0.80** | | **0.44** | | **0.50** | | **-0.28** | | **-0.06** | **-0.78** | |
|  |  | GA_1_ | | **0.37** | **0.62** | | **0.61** | | **0.87** | | **0.40** | | **0.74** | | **1** | | **0.61** | | **-0.03** | | **-0.73** | | **-0.46** | **-0.66** | |
|  |  | Prom/Inh | | **-0.64** | **-0.55** | | **-0.68** | | **-0.62** | | **-0.59** | | **-0.68** | | **-0.46** | | **1** | | **0.08** | | **-0.38** | | -0.02 | **-0.45** | |
|  |  | Glu | | **0.62** | **0.84** | | **0.76** | | **0.86** | | **0.74** | | **0.75** | | **0.66** | | **-0.51** | | **1** | | **0.68** | | **0.73** | **-0.47** | |
|  |  | Fru | | **-0.06** | **0.27** | | **0.03** | | **0.28** | | **0.55** | | **0.03** | | **0.23** | | **-0.13** | | **0.62** | | **1** | | **0.82** | **0.12** | |
|  |  | Sorb | | **0.18** | **0.51** | | **0.30** | | **0.52** | | **0.72** | | **0.30** | | **0.45** | | **-0.34** | | **0.79** | | **0.95** | | **1** | **0.15** | |
|  | | Return bloom | | **-0.06** | **-0.34** | | **-0.13** | | **-0.30** | | **-0.52** | | **-0.13** | | **-0.25** | | **0.10** | | **-0.41** | | **-0.65** | | **-0.65** | **1** | |

Values in bold are different from 0 with a significance level alpha=0.95

|  |  |
| --- | --- |
| **FIGURE S1** The ratio of phytohormones in ʹAuksisʹ apple tree buds grafted on different rootstocks, crop load adjusted to 113 inflorescences per tree during dormancy induction and active bud development.  The data were processed using analysis of variance (Anova). the Turkey (HSD) multiple range test at the confidence level p = 0.05. IAA – indolyl-3 acetic acid; ABA – abscisic acid; GA_3_ – gibberellic acid. | |
